# Collection of *Macaca fascicularis *cDNAs derived from bone marrow, kidney, liver, pancreas, spleen, and thymus

**DOI:** 10.1186/1756-0500-2-199

**Published:** 2009-09-29

**Authors:** Naoki Osada, Makoto Hirata, Reiko Tanuma, Yutaka Suzuki, Sumio Sugano, Keiji Terao, Jun Kusuda, Yosuke Kameoka, Katsuyuki Hashimoto, Ichiro Takahashi

**Affiliations:** 1 Department of Biomedical Resources, National Institute of Biomedical Innovation, 7-6-8 Saito-Asagi, Ibaraki, Osaka 567-0085, Japan; 2 Department of Medical Genome Sciences, Graduate School of Frontier Sciences, University of Tokyo, 5-1-5 Kashiwano-ha, Kashiwa, Chiba 277-8561. Japan; 3 Tsukuba Primate Research Center, National Institute of Biomedical Innovation, 1 Hachimandai, Tsukuba 305-0843, Japan

## Abstract

**Background:**

Consolidating transcriptome data of non-human primates is essential to annotate primate genome sequences, and will facilitate research using non-human primates in the genomic era. *Macaca fascicularis *is a macaque monkey that is commonly used for biomedical and ecological research.

**Findings:**

We constructed cDNA libraries of *Macaca fascicularis*, derived from tissues obtained from bone marrow, liver, pancreas, spleen, and thymus of a young male, and kidney of a young female. In total, 5'-end sequences of 56,856 clones were determined. Including the previously established cDNA libraries from brain and testis, we have isolated 112,587 cDNAs of *Macaca fascicularis*, which correspond to 56% of the curated human reference genes.

**Conclusion:**

These sequences were deposited in the public sequence database as well as in-house macaque genome database . These data will become valuable resources for identifying functional parts of the genome of macaque monkeys in future studies.

## Findings

*Macaca fascicularis *(cynomolgus, crab-eating, or long-tail macaque) is one of the most popular primate species used in biomedical research, and is closely related to *Macaca mulatta *(rhesus macaque). The draft sequence of the *Macaca mulatta *genome, which has an evolutionary important position, was published in 2007 [1].

Transcriptiome data broadens the application of genome sequences. Compared with several millions of human transcript sequences, macaque transcriptome data has only been analyzed in a limited numbers of studies [2-6]. A complete list of macaque genes will be beneficial for performing genetic studies using macaques in the future. We aim to elucidate all the macaque transcripts that correspond to human genes, which have been widely accepted as reference sequences, such as the RefSeq sequences [7].

We have published expressed sequence tag (EST) and full-length sequences, which were obtained from cDNA libraries of brain and testis of *Macaca fascicularis*, using a variety of research subjects [5,8-13]. Here, we present 5'-EST sequences from six other tissues of *Macaca fascicularis*. Bone marrow, liver, pancreas, spleen, and thymus from a 4-year-old male Malaysian *Macaca fascicularis*, and kidney from a 3-year-old female Philippine *Macaca fascicularis *were harvested. These animals are bred and reared in the Tsukuba Primate Research Center (TPRC), National Institute of Biomedical Innovation (Ibaraki, Japan). The tissues were harvested in the P2 facility in TPRC, in accordance with the guidelines of the Laboratory Biosafety Manual, World Health Organization. The libraries for kidney (QreA and QreB) and liver (QlvC) were constructed using the vector-capping method [14], and those for bone marrow (QbmA), pancreas (QpaA), spleen (QspA), and thymus (QthA) were constructed using the oligo-capping method [15]. The sequences of 5'-EST were determined by Sanger sequencing using an ABI 3730 sequencer, and all vector sequences were filtered out [5]. Nucleotide calls with a quality value (QV) of less than 15 were masked as ambiguous. After the masking, the sequences were trimmed, such that they did not contain more than four ambiguous nucleotides in a 10-bp width window, and sequences shorter than 100 bp after the trimming were filtered out. After the trimming, the average sequence length was 886.9 bp.

**Figure 1:**
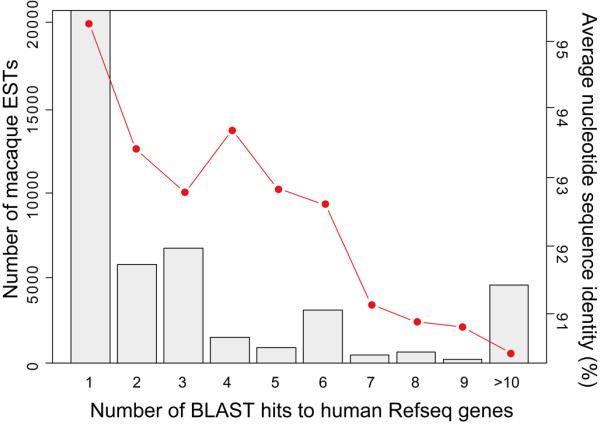
**Number of BLAST hits (cutoff: 1e-60) against the human RefSeq genes**. The grey bars represent the number of macaque ESTs matched to the human RefSeq genes. ESTs matched more than nine RefSeq genes were combined into a single bin. The red circles and lines represent the average nucleotide sequence identity between the macaque ESTs and RefSeq genes, ordered by the rank of BLAST hits. For example, the sequence identity in the second bin represents the sequence identity between the second best hits.

In total, we obtained 56,856 EST sequences from the six tissues. The repeat sequences were masked by Repbase Update before the BLAST search [16]. The BLAST search (BLASTN) was performed with a cut-off value (*E*-value) of 1e-60 against human RefSeq data [7]. Since RefSeq sequences contain partially overlapped isoforms, we constructed non-redundant RefSeq sequences based on the Entrez Gene database [17]. Hereafter, we shall refer to the non-redundant RefSeq sequences as RefSeq genes. There were 23,236 RefSeq genes, including non-coding RNAs in the human genome at the time of investigation (Release 34) [7]. Out of the newly isolated 56,856 cDNA clones, 44,603 matched to 4940 human RefSeq genes. Of the 12,253 non-RefSeq clones, 40 consisted of repeat sequences, and the other 1631 did not show any homology to human transcript sequences in public databases using a lower cutoff value (1e-15). Meanwhile, 23,900 EST sequences were homologous to multiple RefSeq genes with the high cutoff value (1e-60). The average nucleotide sequence identity between the best BLAST hit pairs was 95.26%. The nucleotide sequence identity was slightly lower than that estimated using full-length cDNA sequences of high quality [5], and supposed to reflect some sequencing errors in the EST sequences. In some cases, the nucleotide sequence identity between the best and second best hit pairs were very close, which was probably due to gene duplications specific in the human lineage. The difference in nucleotide sequence identities between the best and second best BLAST hits were less than 0.5% in 8996 ESTs. In such cases, the best hit orthologs would not be regarded as unique orthologs of humans and macaques. In Figure 1, we classify the macaque ESTs according to the number of BLAST hits to RefSeq genes. The average nucleotide sequence identities were ordered by the rank of BLAST hits. For example, the nucleotide sequence identity in the second bin represents the identity between the second best hit pairs.

In conjunction with the previously sequenced cDNA clones, we obtained 112,587 EST sequences corresponding to 8262 human RefSeq genes, which correspond to 36% of all human RefSeq genes. When we restricted the analysis of the human RefSeq genes in the manually curated status (Reviewed or Validated status) [7], 56% (6,177/11,080) of the human RefSeq genes were covered by the macaque transcriptome.

As shown in Table 1, the number of RefSeq genes that were represented in the libraries was different in different tissues. In order to measure the unbiased transcript redundancy in each tissue, we estimated the redundancy of the human RefSeq homologs in 1000 macaque transcripts in each tissue. We randomized the transcript data and selected 1000 transcripts to enumerate the human RefSeq genes covered by the transcripts. The redundancy was given by the number of transcripts (1000) divided by the number of human RefSeq genes covered by the transcripts. This procedure was repeated 1000 times for each tissue, and the average redundancy was estimated. The results are shown in the last column of Table 1. Pancreas showed the highest redundancy; while brain and testis showed low redundancy, indicating that the gene expression complexity in brain and testis is higher than that in the other tissues, as suggested previously [18]. We also found that the kidney library (QreA) had very low redundancy. It was constructed using the vector-capping method, which does not amplify the template cDNA by PCR and may reduce the redundancy of the library [14]. In order to test the effectiveness of the cloning methods, we compared the redundancy of the transcript in our liver library constructed using the vector-capping method, and the previously reported liver library constructed using the oligo-capping method [6]. The redundancy in the vector-capped liver library was 3.21 (Table 1). In contrast, the redundancy in the oligo-capped liver library was 5.19 [6], which was significantly higher than that in the vector-capped library (*P *< 0.001, permutation test).

**Table 1:** Summary of *Macaca fascicularis *cDNA libraries

**Tissue**	**Total clones**	**Covered RefSeq^d^**	**non-RefSeq^e^**	**Redundancy^f^**
Brain cortex^a, c^	28679	4035	10259	2.32
Brain stem^b, c^	5758	1591	2050	2.40
Cerebellum^c^	11003	2340	4179	2.32
Testis^c^	8551	1833	3300	2.36
Liver	9188	1360	3853	3.21
Kidney	9558	2495	2630	1.91
Bone marrow	9472	1366	1317	3.26
Spleen	9783	1556	1527	3.15
Thymus	9566	1295	1491	2.96
Pancreas	9289	534	1435	9.83
All	112587	8262	32269	2.14

^a^Brain cortex includes parietal lobe (Qnp), temporal lobe (Qtr), occipital lobe (Qor), and frontal lobe (Qfl).

^b^Brain stem includes medulla oblongata (Qmo) and the other part of brain stem (Qbs).

^c^These sequences were determined by the previous studies [8-10,12].

^d^Number of human RefSeq genes that have macaque homologs in each library.

^e^TheNumber of macaque cDNA clones that do not have human RefSeq homologs.

^f^Estimated from randomly chosen 1000 macaque transcripts, averaged over 1000 simulations.

We have developed an in-house database for the genome data of *Macaca fascicularis *(QFbase: ) [5]. The *Macaca fascicularis *cDNA sequences described in this report were annotated and added to this database. They were also mapped on the rhesus macaque genome sequence using the BLAT program [19]. The results can be viewed in the *Macaca fascicularis *genome browser , which is implemented using GBrowse software [20]. The DDBJ/EMBL/Genbank accession numbers of these sequences are DC629777-DC639249 (bone marrow), DC639249-DC648806 (kidney), DC620589-DC629776 (liver), FS362802-FS372090 (pancreas), DC848487-DC858269 (spleen), and DK575154-DK584719 (thymus).

## Availability and requirements

• **Project name**: *Macaca fascicularis *cDNA sequencing project

• **Project home page**: 

• **Operating system(s)**: Platform independent

• **Programming language**: PERL

• **Other requirements**: Generic web browser

• **License**: GNU, GPL

• **Any restrictions to use by non-academics**: none

## Abbreviations

EST: expressed sequence tag; QV: quality value;

## Competing interests

The authors declare that they have no competing interests.

## Authors' contributions

NO, KT, JK, YK, KH, and IT contributed to the design of the research. NO analyzed the data. NO and KH wrote the manuscript. MH performed the computational analysis. RT, YK, and IT were involved in the cDNA sequencing. YS and SS constructed the oligo-capped cDNA libraries. All authors read and approved the final manuscript.
